# Three-dimensional feature matching improves coverage for single-cell proteomics based on ion mobility filtering

**DOI:** 10.1016/j.cels.2022.02.003

**Published:** 2022-03-16

**Authors:** Jongmin Woo, Geremy C. Clair, Sarah M. Williams, Song Feng, Chia-Feng Tsai, Ronald J. Moore, William B. Chrisler, Richard D. Smith, Ryan T. Kelly, Ljiljana Paša-Tolić, Charles Ansong, Ying Zhu

**Affiliations:** 1 Environmental Molecular Sciences Laboratory, Pacific Northwest National Laboratory, Richland, WA 99354, USA; 2 Biological Sciences Division, Pacific Northwest National Laboratory, Richland, WA 99354, USA; 3 Department of Chemistry and Biochemistry, Brigham Young University, Provo, UT 84602, USA; 4 These authors contributed equally; 5 Lead contact

## Abstract

Single-cell proteomics (scProteomics) promises to advance our understanding of cell functions within complex biological systems. However, a major challenge of current methods is their inability to identify and provide accurate quantitative information for low-abundance proteins. Herein, we describe an ion-mobility-enhanced mass spectrometry acquisition and peptide identification method, transferring identification based on FAIMS filtering (TIFF), to improve the sensitivity and accuracy of label-free scProteomics. TIFF extends the ion accumulation times for peptide ions by filtering out singly charged ions. The peptide identities are assigned by a three-dimensional MS1 feature matching approach (retention time, accurate mass, and FAIMS compensation voltage). The TIFF method enabled unbiased proteome analysis to a depth of >1,700 proteins in single HeLa cells, with >1,100 proteins consistently identified. As a demonstration, we applied the TIFF method to obtain temporal proteome profiles of >150 single murine macrophage cells during lipopolysaccharide stimulation and identified time-dependent proteome changes. A record of this paper’s transparent peer review process is included in the supplemental information.

## In brief

Single-cell proteomics (scProteomics) holds great potential to advance our understanding of the heterogeneity of cellular phenotypes. Because of the lack of global protein amplification approaches, scProteomics is challenged to characterize low-abundance proteins, which are usually involved in critical biological processes. We develop an MS1-centric data acquisition and peptide identification method, transferring identification based on FAIMS filtering (TIFF), that improves proteome coverage, quantification accuracy, and throughput of label-free scProteomics. We demonstrated that the improved scProteomics platform can reveal the proteome changes of macrophage activation and classify dissociated human lung cells into distinct populations.

## Graphical Abstract

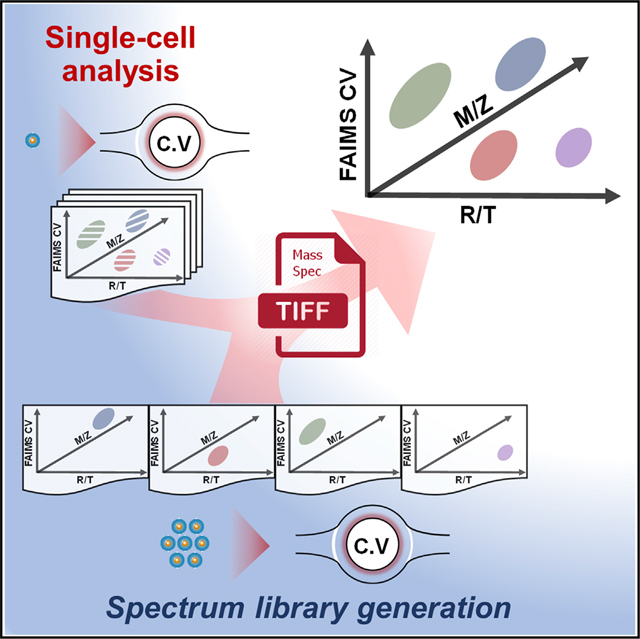

## INTRODUCTION

Single-cell technologies have become the cornerstone of biomedical and cell biology research (Hou et al., 2016; Wilson et al., 2015). The emergence of single-cell RNA sequencing (scRNA-seq) and related single-cell sequencing technologies has illuminated unappreciated cellular heterogeneity and revealed cell subpopulations obscured in bulk measurements (Stuart and Satija, 2019). However, many integrative studies have shown only low-to-moderate correlations between the abundance of RNA transcripts and their corresponding proteins (Du et al., 2019; Qian et al., 2016), as the translation of RNA into a functional protein can be affected by diverse events such as alternative splicing and microRNA regulation (Buettner et al., 2015). Additionally, RNA measurements cannot infer post-translational modifications that modulate protein functions. Thus, there is an unmet need for broad proteome measurements at the single-cell level, which has lagged behind single-cell sequencing approaches.

Recent advances in sample preparation and mass spectrometry facilitate unbiased single-cell proteomics (scProteomics) (Budnik et al., 2018; Cheung et al., 2021; Cong et al., 2020; Dou et al., 2019; Hartlmayr et al., 2021; Li et al., 2018; Schoof et al., 2021; Shao et al., 2018; Specht et al., 2021; Tsai et al., 2020; Williams et al., 2020; Woo et al., 2021; Zhu et al., 2018a, 2018b, 2019). Microfluidic sample processing devices and systems have improved protein digestion efficiency and minimized adsorptive sample losses (Hartlmayr et al., 2021; Li et al., 2018; Shao et al., 2018; Woo et al., 2021; Zhu et al., 2018a, 2018b). Tandem mass tag (TMT)-based isobaric labeling approaches (e.g., ScoPE-MS) have enabled multiplexed single-cell analysis in individual LC-MS runs (Budnik et al., 2018; Dou et al., 2019; Hartlmayr et al., 2021; Schoof et al., 2021; Specht et al., 2021; Tsai et al., 2020). The miniaturization of capillary electrophoresis or liquid chromatography has improved separation resolution and enhanced electrospray ionization efficiency (Xiang et al., 2020). High-resolution MS analyzers combined with ion focusing devices, such as ion funnel, have increased detection sensitivity to the level where single molecules can be detected (Makarov and Denisov, 2009). State-of-the-art methodologies in scProteomics now can identify from ∼700 to ∼1,000 proteins from cultured single mammalian cells (e.g., HeLa) using label-free approaches (Cong et al., 2020, 2021; Williams et al., 2020; Zhu et al., 2018a) and from ∼750 to ∼1,500 proteins using TMT-labeling strategies (Budnik et al., 2018; Dou et al., 2019; Schoof et al., 2021; Specht et al., 2021; Tsai et al., 2020; Woo et al., 2021). Despite these advances, scProteomics remains immature, and technical challenges remain, including not only limited proteome depth and poor quantification performance but also low system robustness for large-scale single-cell studies. The inability to characterize low-abundance proteins impedes the study of critical biological processes in single cells, such as signal transduction and gene regulation.

Because of the lack of a global amplification method for proteins, the coverage and quantification performance of scProteomics largely rely on the capabilities of MS measurement (e.g., sensitivity, speed, and dynamic range). Although targeted MS measurements enable the detection of low copy number proteins and even single molecules (Makarov and Denisov, 2009), these measurements are generally performed using narrow *m*/*z* windows (Makarov and Denisov, 2009) or tandem mass spectra (Amenson-Lamar et al., 2019) to minimize background signals. Background ions, originating from ambient air and solvent/reagent impurities, dominate MS spectra during full *m/z* range acquisition. These abundant ions quickly fill ion trapping devices (e.g., ion trap or ion routing multipole) and limit the ability to trap ions over an extended time, which could otherwise accumulate low-abundance ions of interest and improve detection sensitivity (Meier et al., 2018; Pasa-Tolić et al., 2002). The high background signals can also reduce the dynamic range of MS analyzers and deteriorate feature detection during downstream data analysis.

We reasoned that the removal of background ions should dramatically enhance the sensitivity of MS detection and improve the proteome coverage and quantification performance of scProteomics. A variety of approaches have been developed to minimize background signals, including the use of a carbon filter in front of MS inlets to purify the ambient air (Luber et al., 2010), a picoliter-flow liquid chromatography (LC) system to reduce overall contaminates from air and solvent (Xiang et al., 2020), a dynamic range enhancement applied to MS (DREAMS) data acquisition algorithm to reject highly abundant ions before ion accumulation (Pasa-Tolić et al., 2002), and a high field asymmetric waveform ion mobility spectrometry (FAIMS) interface to remove singly charged ions (Cong et al., 2020). Recently, Cong et al. (2020) demonstrated the coupling of FAIMS with low-flow LC (20 nL/min), and Orbitrap Eclipse can identify ∼1,100 proteins from single cells. Because the peptides were identified by MS/MS, long LC gradients were required to collect sufficient numbers of MS/MS spectra for deep proteome coverages, which limited analysis throughput. Herein, to address these challenges, we describe an MS1-centric data acquisition and peptide identification method, transferring identification based on FAIMS filtering (TIFF), that improves the proteome coverage, quantification accuracy, and throughput of label-free scProteomics. We demonstrated the capability and scalability of the TIFF method by studying macrophage activation with lipopolysaccharide (LPS) and by classifying dissociated human lung cells into distinct populations.

## RESULTS

### The TIFF method

**Figure 1.**
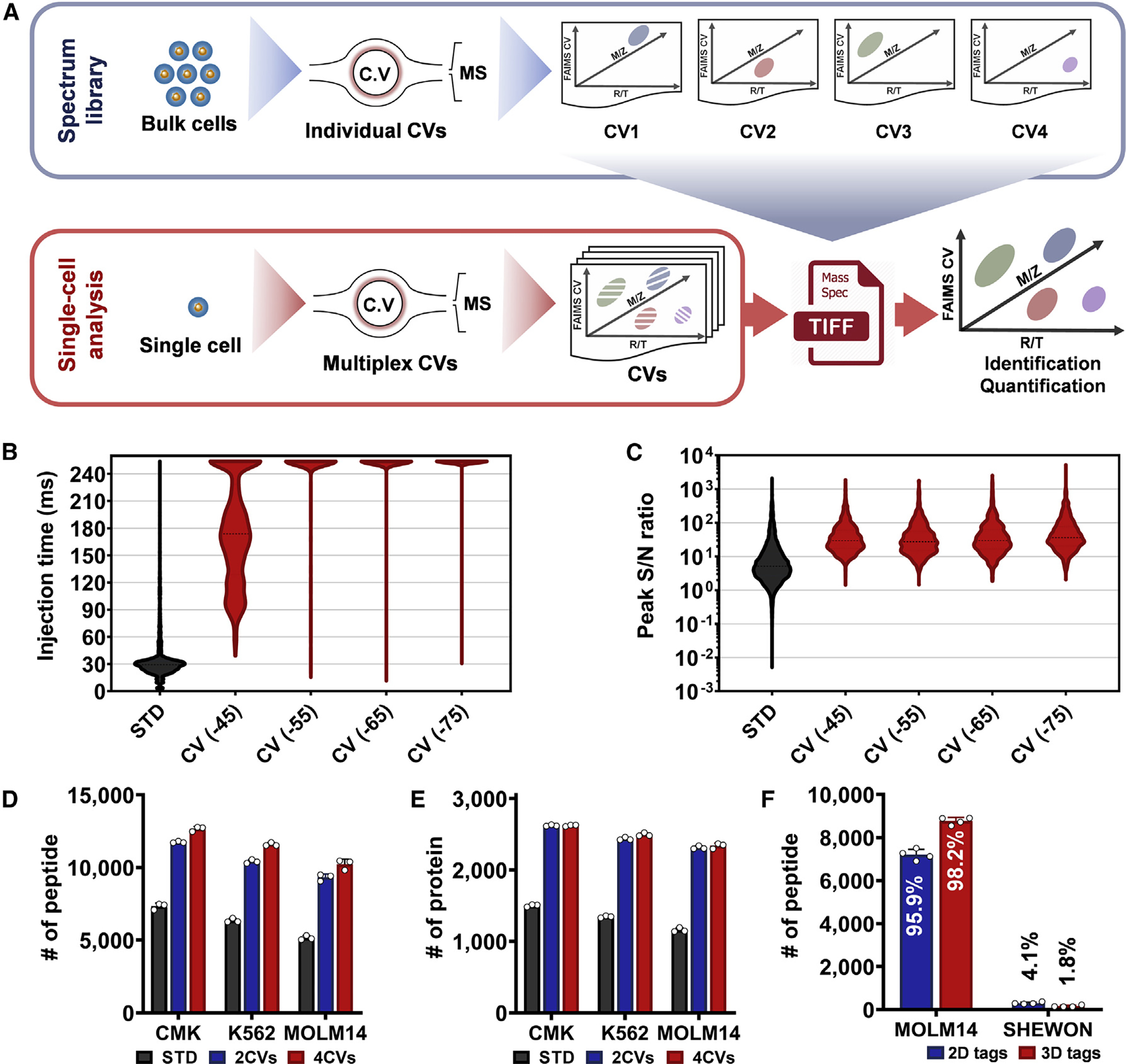
The concept of the TIFF method (A) Workflow of the transferring identification based on the FAIMS filtering (TIFF) method. High-input samples (usually from 50 to 100 cells) are analyzed by LC-FAIMS-MS with each LC-MS analysis utilizing a discrete FAIMS CV to generate a spectral library. Single-cell samples are analyzed by cycling through multiple FAIMS CVs for each LC-MS analysis. Peptide features in single cells are identified by matching to the spectral library based on three-dimensional (3D) tags, including LC retention time (RT), *m/z*, and FAIMS CV. (B) MS1 injection time (IT) distributions for single-cell level peptides (0.2 ng, CMK cell) in the standard (STD, no FAIMS) method and FAIMS method with four different CVs. The numbers (*n*) of IT data points are 5,525 in STD and 1,774 in each FAIMS CV. (C) The distributions of signal-to-noise ratios (S/N) of LC-MS features for the 0.2-ng peptides in STD run and FAIMS run with 4 CVs. The numbers (*n*) of S/N data points are 10,232 in STD run, 1,546 in CV −45, 1,539 in CV −55, 1,348 in CV −65, and 1,358 in CV −75. (D) The average number of unique peptides and (E) the corresponding unique proteins using single-cell level (0.2 ng) protein digests from three cell lines (CMK, K562, and MOLM14). Benchmarking analysis was performed with the STD, 2-CV TIFF (−45 and −65 V), and 4-CV TIFF (−45, −55, −65, and −75 V) methods. The data point (n) to generate the bar graphs is 3. (F) The number of human peptides (MOLM-14) and bacterial peptides (SHEWON) identified from 2D and 3D tag methods. See also Figure S4. The bacterial peptides were considered false identifications. The data point (n) to generate the bar graphs is 4. The error bars in (D–F) represent standard deviations (SDs).

The TIFF method is inspired by the accurate mass and time (AMT) tag approach (Pasa-Tolić et al., 2004) or other derivative approaches, such as "match between run" (MBR), implemented in MaxQuant (Tyanova et al., 2016a) or IonQuant (Yu et al., 2021), that generally rely on two measurements for the assignment of peptide identity: the accurate mass-to-charge ratio (*m/z*) and the LC retention time (RT). We have previously demonstrated that MBR improves the proteome coverage and reduces missing values in scProteomics (Zhu et al., 2018b). The recent integration of ion mobility devices, including FAIMS at the interface between the LC system and mass spectrometer, provides an opportunity to use the additional ion mobility separation dimension to reduce false discovery rate (FDR) and improve coverage (Prianichnikov et al., 2020). We take advantage of this advance and utilize the FAIMS compensation voltage (CV) as a third matching feature (in addition to retention time and accurate mass) for peptide identification, as illustrated in Figure 1A. Briefly, a spectral library is constructed by repeatedly analyzing high-input samples on an LC-FAIMS-MS platform, with each LC-MS analysis utilizing a discrete FAIMS CV. Each peptide identified in the high-input analyses is associated with a unique 3-dimensional (3D) tag comprising LC retention time, accurate *m/z*, and FAIMS CV. Next, low-input samples (e.g., single cells) are analyzed by cycling through multiple FAIMS CVs within a single LC-MS analysis. A key aspect of the TIFF method is the mode of MS data acquisition, with most of the MS time spent on MS1 acquisition to enhance the accumulation of low-abundance peptide ions for sensitive detection. Compared with our previous FAIMS-based scProteomics method (Figures S1A and S1B), precursor ion sampling time is increased by >3-fold (Figure S1C). The fewer MS2 acquisitions generated within each cycle are sufficient to exploit the nonlinear multisample alignment feature of MaxQuant. Subsequently, MS1 features in low-input samples (i.e., single cells) are identified by matching to the spectral library and utilizing the unique 3D tag based on the MBR algorithm within MaxQuant (Tyanova et al., 2016a).

### TIFF improves LC-MS sensitivity

We first verified the utility of FAIMS to remove singly charged ions (“chemical background” noise) and create more “room” for peptide ion accumulation to enhance detection of low-abundance peptides. We analyzed single-cell equivalent amount (0.2 ng) of protein digests (CMK, human acute megakaryocytic leukemia cells) with or without a FAIMSpro interface. Without FAIMS, most dominating signals corresponded to singly charged ions, some of which are known to originate from plasticizers (e.g., *m/z* 391.28) and air impurities (e.g., *m/z* 445.12, 462.29, and 519.14) (Figure S2). Because these highly abundant contaminants quickly filled ion accumulation (or trapping) regions, the median ion injection/accumulation time was only 30 ms across the whole LC-MS analysis (Figure 1B). In comparison, when FAIMS was used, most dominating ion signals were multiply charged (Figure S2), and the median ion injection times increased from ∼30 to ∼180 ms for a CV of −45 V, reaching a maximal time of 254 ms for the other three CVs. This corresponded to an ∼8.53× increase in ion accumulation time (Figure 1B). Benefiting from the low background and elongated ion accumulation, the median S/N of LC-MS features increased from 5.2 (STD) to 29.6 (FAIMS), representing a >5-fold increase for all the CVs (Figure 1C).

To evaluate the improvements in MS sensitivity, we investigated several metrics related to proteome coverage, including the number of multiply charged MS features, unique peptides, and proteins (Figures 1D, 1E, and S3A–S3D). Briefly, we analyzed single-cell-level (0.2 ng) protein digests from three leukemia cell lines: CMK, K562, and MOLM14 with either a FAIMSpro interface or with a standard interface. Compared with the standard interface, the FAIMSpro interface and the TIFF method increased the number of multiply charged MS features detected in the MS1 by >3-fold (Figure S3A). Most of the increased peptide features appeared in the low-MS-intensity scale across all four FAIMS CVs (Figure S3E). Similarly, the TIFF method increased peptide identification by >75% (Figure 1D) and protein identification by >74% (Figure 1E). As expected, the MS/MS-based identifications were reduced due to the lower number of MS/MS scans (Figures S3B–S3D) in the TIFF method. Modulation of CVs within the TIFF method had a modest effect on the number of peptide features, peptides, and proteins, with only a slight increase using 4 CVs as opposed to 2 CVs. However, utilizing 4 CVs in the TIFF method yielded increases in summed peptide intensities compared with utilizing 2 CVs (Figure S3F), which subsequently improved the quantification performance as described below.

We evaluated whether the 3D feature matching approach could reduce FDR by comparing it with the conventional 2D matching approach (Pasa-Tolić et al., 2004; Tyanova et al., 2016a). We generated a mixed-species spectral library containing 20,588 human peptides from MOLM14 cells and 9,362 bacterial peptides from *Shewanella Oneidensis* MR-1 (SHEWON). These *Shewanella* proteins were served as “decoy” proteins in the library. To do this, we analyzed 0.1-ng MOLM14 peptides with the 4CV-FAIMS method. During MaxQuant analysis with MBR algorithm, we either disabled or enabled the FAIMS CV matching function. As shown in Figures 1F and S4, the conventional 2D matching approach resulted in a total of 7,199 peptides identified, and 304 of them were bacterial peptides, representing a false matching rate of 4.1%. Encouragingly, when the 3D matching approach (TIFF) was applied, only 161 bacterial peptides were identified, corresponding to a false matching rate of 1.8%. At the protein level, the FDRs of 2D and 3D matching approaches were estimated to be 10.8% and 5.3% (Figure S4D), respectively.

### TIFF improves the quantification of scProteomics

Next, we evaluated whether the TIFF method improves quantification performance when compared with a standard approach. We compared the run-to-run reproducibility from triplicates using 0.2 ng of CMK cell digests with the standard, 2-CV TIFF, and 4-CV TIFF methods. Although the distribution of the coefficients of variation was similar between the 2-CV TIFF and the standard methods, the median of the coefficients of variation for the 4-CV TIFF method was significantly reduced from 15.6% to 12% (Figure S5A). Such an improvement could be attributed to the enhanced sensitivity of the 4-CV TIFF method, allowing more low-abundance peptides to be identified. With the 4-CV TIFF method, >80% of the proteins had no missing values and >90% had no more than one missing value across the triplicates. Higher percentages of missing data were present with the 2-CV TIFF and standard methods (Figure S5B). To further assess the quantification accuracy of the 4-CV TIFF method, we performed a statistical analysis using samples from two cell types (CMK and K562). Proteins having at least 2 valid values in a given group were considered quantifiable. The 4-CV TIFF method exhibited a total of 2,345 quantifiable proteins that included ∼98% of the proteins (1,052) using the standard method (Figure S5C). Because it was possible to quantify proteins more consistently with the TIFF method, we observed 1,053 differentially abundant proteins (DAPs) (FDR < 0.05 and S_0_ = 0.1) between the CMK and K562 cells, whereas only about half (i.e., 536 DAPs) were found using the standard method (Figures S6A and S6B). A total of 380 DAPs were shared between the two methods. As shown in Figure S5D, the linear correlation coefficient of protein fold changes between the two label-free methods was high (R = 0.95). The slope of linear regression was ∼1 (*K*), indicating similar fold changes between the two methods. Similarly, the 4-CV TIFF method showed improved quantification results over the standard method in the comparison between MOLM14 and the other two cell types (Figures S7 and S8).

### A streamlined label-free scProteomics platform

Having demonstrated that the TIFF method offers improvements in proteome coverage and quantification for mass-limited samples, we integrated it into our scProteomics pipeline that includes fluorescence-activated cell sorting (FACS) for cell isolation (Zhu et al., 2018a), a robotically addressed nanowell chip for single-cell processing nanodroplet processing in one pot for trace samples (nanoPOTS) (Zhu et al., 2018b), a nanoliter-scale LC autosampler for reliable sample injection (Williams et al., 2020), and a low-flow LC system (LC column with 50 μm i.d.) (Williams et al., 2020). Both single cells and pooled library cells can be isolated with FACS and processed with nanoPOTS. The integrated FACS-nanoPOTS-autosampler-TIFF-MS platform offered a complete solution from cell isolation to data acquisition and peptide identification for unbiased scProteomics, as well as other biological applications with mass-limited samples. The platform is robust and scalable. Since developed, it has been used to analyze >1,200 samples in our facility.

### Proteome coverage of single HeLa cells

We used HeLa cells to benchmark the TIFF-based scProteomics workflow. Using a tandem mass spectrometry approach (MS/MS), an average of 209 proteins were identified from single HeLa cells (Figure S9A). The number was comparable with our previously reported result (211 proteins) using a lower-flow LC-MS system (50 nL/min with 30 μm i.d. column) but without a FAIMS interface (Table S1) (Zhu et al., 2018a), and 42% lower than that obtained using an ultralow-flow LC system (20 μm i.d. column) and the newest generation (Eclipse) MS (Cong et al., 2020). The utilization of the 4-CV TIFF method dramatically increased the coverage to an average of 1,212 (±10%) identified protein across 10 single cells (Figure S9A). The TIFF method doubled the total number of identifications compared with our previous report (Zhu et al., 2018a), reaching 1,771 unique proteins (Figure S9B). The number of identifications obtained with the TIFF method was comparable with the one that we obtained using a 20-μm-i.d. column (20 nL/min), a FAIMS interface, an Eclipse MS, and a long LC gradient (Cong et al., 2020).

The quantification consistency was also evaluated. Using protein iBAQ intensities, 684 of 1,771 proteins had no missing values across the 10 HeLa cells (Figure S9C). In total, 1,103 proteins were presented in at least 50% of the analyses. Pearson’s correlation coefficients had a median value of 0.95 between any two HeLa cells, indicating the high reproducibility of our integrated scProteomics pipeline (Figure S9D). Together, these results demonstrated that the integration of the TIFF method with high-efficiency single-cell preparation offers a sensitive and reliable scProteomics pipeline for label-free quantification.

### Preliminary application to dissociated primary cells from human lung

To initially explore the scProteomics platform for cell-type classification from dissociated primary cells, we analyzed nondepleted and nonlabeled primary cells from the lung of a 2-year-old donor (Figure S10A). In total, 19 single cells were processed and analyzed using the TIFF-based scProteomics workflow, resulting in a total of 986 identified proteins with an average of 390 identified proteins per single cell (Table S2). We retained proteins identified in at least 8 of the 19 single cells (40% presence) for quantitative analysis, resulting in 402 quantifiable proteins (Table S3). Principal component analysis (PCA) of the 402 proteins suggested the presence of at least three cell populations in the lung tissue single-cell suspension (Figure S10B).

To identify proteins distinguishing these populations, we performed the ANOVA test (permutation-based FDR < 0.05, S_0_ = 0), revealing 99 proteins (∼20% of quantifiable proteins) that were differentially abundant across the three cell population groups/clusters (Table S4) as visually represented in Figure S10C. Cell-type identity was assigned to each of the three cell population groups by comparing markers from the scProteomics data with lung cell-type markers previously enumerated by bulk proteomics of sorted cell populations of human lung endothelial, epithelial, immune, and mesenchymal cells (Du et al., 2019). Correspondence analysis across the scProteomic and bulk proteomic markers revealed that Cluster 1 represented a lung endothelial cell population, Cluster 2 represented a lung immune cell population, and Cluster 3 represented a lung epithelial cell population (Figure S11). For example, Caveolin-1 (CAV1) and Polymerase I and transcript release factors (PTRF), which were highly abundant in single-cell cluster 1 (Figures S10D and S11), were known to structurally maintain the specialized lipid raft of caveola in lung endothelial cells (Chettimada et al., 2015). L-Plastin (LCP1) protein, important for alveolar macrophage development and antipneumococcic response (Deady et al., 2014), was highly abundant in bulk sorted immune cells as well as Cluster 2. Pulmonary surfactant-associated protein B (SFTPB), which facilitates alveolar stability by modulating surface tension (Wang et al., 2018), was known to be preferentially enriched in lung epithelial cells. SFTPB was highly abundant in bulk sorted epithelial cells as well as Cluster 3. The above results demonstrated the feasibility of the scProteomics platform for cell-type classification from nondepleted whole tissue single-cell suspension samples.

We also examined the abundance patterns of the 17 marker proteins based on scProteomics, bulk proteomics, and transcriptomics of the sorted populations (Figure S11). For the three protein markers mentioned above, we observed good agreement in all three measurement modalities. However, similar to our previous integrative study (Du et al., 2019), we observed disagreement for some protein markers. For example, TUBB protein was identified as an endothelial cell marker in the proteomics dataset, but it was not significant in the transcriptomics dataset. In addition, among the 7 epithelial cell markers, only 1 protein/gene (SFTPM) was significantly expressed in both proteomics and transcriptomics datasets.

### Large-scale proteome profiling of single macrophage cells in response to lipopolysaccharide treatment

**Figure 2.**
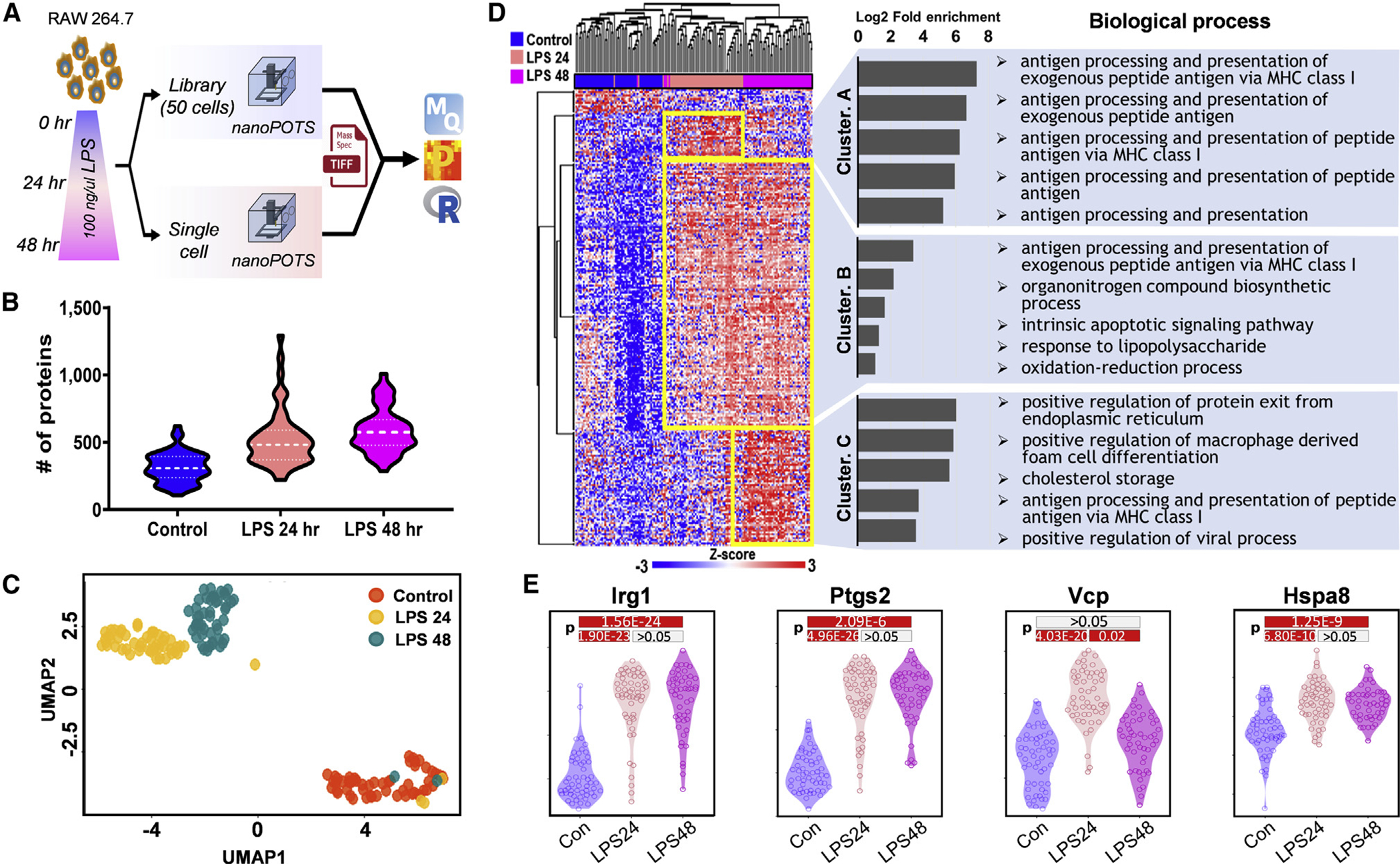
Single-cell proteomics analysis of time-dependent macrophage activation (A) Illustration of workflow for scProteomics analysis of 155 macrophages containing untreated (control) cells and the cells treated by LPS for 24 and 48 h. (B) The distribution of protein identification numbers for each treatment group. (C) The clustering of the 155 single macrophage cells based on treatment groups with UMAP projection, generated by an R package of RomicsProcessor v1.1.0 (https://github.com/PNNL-Comp-Mass-Spec/RomicsProcessor). Source data are provided in Table S5. (D) Heatmap showing the protein abundance differences across the 155 macrophage cells after statistical test using ANOVA (FDR < 0.001, S0 = 5). The hierarchical clustering was performed using the Euclidean method for 250 DAPs by ANOVA test. Functional enrichment analysis was performed with DAVID bioinformatics tools (Huang et al., 2009). The scale bar shows the linear distribution of *Z* scores. (E) Abundance distributions of representative regulated proteins from different treatment conditions. In (B) and (E), the numbers (n) of data points to generate violin plots are 54 for control cells, 52 for LPS 24 h cells, and 49 for LPS 48 h cells.

To further evaluate our platform for large-scale scProteomics analysis, we profiled proteome changes of single murine macrophage cells (RAW 264.7) after 24 and 48 h lipopolysaccharide (LPS) stimulation relative to unstimulated cells (control) (Figure 2A). We analyzed a total of 155 individual RAW 264.7 cells, containing 54 unstimulated cells, 52 stimulated cells at 24 h, and 49 stimulated cells at 48 h. Our analysis identified a total of 1,671 proteins across the 155 individual cells. The median number of proteins identified per cell was 451. Although lower than the number of proteins identified from single HeLa cells described above, we noted that RAW 264.7 cells have a median diameter of 10 μm (Saxena et al., 2003) compared with ∼17 μm for HeLa cells (Zhu et al., 2019); the 5-fold difference in cell volume likely accounted for the reduced coverage. We also observed control cells to have fewer identified proteins than LPS-stimulated cells. The median numbers of identified proteins were 307, 482, and 575 for control, 24 h stimulation, and 48 h stimulation, respectively (Figure 2B). Previous reports have indicated that stimulated RAW 264.7 macrophages increased in size and changed morphology upon LPS stimulation, potentially accounting in part for the difference in identifications (Saxena et al., 2003). Of the 1,671 identified proteins, 519 were conservatively retained for quantitative analysis after filtering out proteins containing >50% missing values in at least one experimental condition (Table S4). Using a uniform manifold approximation and projection (UMAP)-based dimensional reduction analysis (Becht et al., 2019), the 155 individual cells partitioned into three distinct clusters on a two-dimensional plot correspond to the three experimental conditions (Figure 2C). Five stimulated cells (3 from 24 h and 2 from 48 h) were clustered into the control group, indicating that only a small portion of RAW cells (∼5%) is not sensitive to LPS stimulation.

To identify the DAPs that drove the partitioning of the three clusters, we performed an ANOVA test analysis (permutation-based FDR < 0.001, S_0_ = 5). A total of 250 proteins were significantly modulated across the three groups (Figure 2D). Gene ontology analysis results showed that proteins increased in abundance at 24 h LPS stimulation (cluster A in Figure 2D) were primarily enriched in antigen processing and presentation processes (FE = 37.2–157.5, p < 0.01). Proteins increased at 24 h LPS stimulation and remained elevated through 48 h LPS stimulation (cluster B) were enriched in antigen processing and presentation (FE = 10.6, p < 0.05), response to LPS (FE = 2.5, p < 0.05), and oxidation-reduction (FE = 2.1, p < 0.01) processes, which were known to be a critical function of activated macrophage cells. Biological processes enriched in proteins increased after 48 h LPS stimulation (cluster C) included those related to protein exit from the endoplasmic reticulum (FE = 61.8, p < 0.05) and to foam cell differentiation (FE = 56.1, p < 0.05). The latter finding was in line with a previous report on the ability of LPS-activated RAW 264.7 macrophages to differentiate into foam cells (Funk et al., 1993). Proteins associated with cholesterol storage (FE = 47.5, p < 0.05) were also increased in abundance after 48 h LPS stimulation. Storage of cholesterol ester or triglyceride has been suggested to lead to the formation of foam cells (Feingold et al., 2012).

Beyond functional enrichment analysis, our statistical analysis identified specific proteins previously described as being involved in the response process of macrophage cells to LPS stimulation. For example, immune responsive gene 1 (Irg1), known as a resistance-inducing protein against LPS (Li et al., 2013), was upregulated in macrophage cells exposed to LPS at both 24 and 48 h (Figure 2E). Irg1 is highly expressed during various infections or TLR ligand stimulation in macrophages, which have been reported to regulate macrophage innate immune responses by controlling proinflammatory cytokines (Li et al., 2013). Prostaglandin-endoperoxide synthase 2 (Ptgs2/Cox-2), an important precursor of prostacyclin enzyme which is expressed in macrophages exposed to LPS (Tang et al., 2017), was also significantly increased in LPS-stimulated macrophage cells (Figure 2E). Transitional endoplasmic reticulum ATPase (Vcp, also called p97) is involved in the targeting and translocation of ubiquitinated proteins, and the regulation of the inflammatory response in immune cells (Fenech et al., 2020). We observed increased abundances of Vcp at 24 h LPS stimulation with a decrease to basal level at 48 h LPS stimulation. The perturbation of cellular ubiquitin homeostasis supports the concept that variations in protein ubiquitination may be the key response to pathogen infection and trigger the defense mechanism of macrophages. Heat shock cognate 71-kDa protein (Hspa8), known to be involved in the presentation of antigenic peptides by major histocompatibility complex (MHC) class II (MHCII) molecules for CD4 + T cells, was significantly increased in LPS-stimulated cells in line with previous studies that also showed this protein to be overexpressed in response to LPS stimulation (Zhang et al., 2011).

## DISCUSSION

In this study, we developed an ion mobility-enhanced MS acquisition and peptide identification method, TIFF (Transferring Identification based on the FAIMS Filtering), which was coupled with our previously described nanoPOTS scProteomics workflow (Williams et al., 2020; Zhu et al., 2018b) to improve the sensitivity and accuracy of label-free scProteomics. MS acquisition efficiency was improved by filtering out singly charged background ions and allowing ion accumulation for extended periods for sensitive detection. Compared with our previous FAIMS-based scProteomics workflow using an ultralow-flow LC column (20-μm-i.d.) and long gradient,^12^ the TIFF method dramatically improved both system robustness and analysis throughput to enable large-scale single-cell studies. The TIFF-based workflow enabled the identification of >1,700 proteins and quantification of ∼1,100 proteins from single HeLa cells with label-free analysis. We demonstrated the robustness and scalability of the scProteomics workflow via a large-scale analysis of 155 single macrophage cells under different LPS stimulation conditions to reveal the biological processes at the single-cell level. Finally, we demonstrated the feasibility of classifying cell populations of a human lung.

Although our label-free analysis of single cultured cells (e.g., HeLa) yielded >1,000 proteins identified and a similar number of proteins quantified, a similar analysis of single primary cells (e.g., human lung cells) resulted in significantly fewer proteins, presumably due to the fact that culture cells have larger sizes and more proteins mass (Figure S12). This again highlights the need to further improve the overall sensitivity of current scProteomics platforms to enable routine and deep single-cell proteome analyses of primary cells derived from tissues of animal models and human donors. One strategy for improving overall sensitivity is to further improve protein/peptide recovery. Sample recovery during sample processing procedures could be increased using smaller nanowells or low-binding surfaces to reduce adsorptive loss. Another strategy for improving overall sensitivity is through enhancing peptide separation resolution and ionization efficiency. With the advances of nanoLC pump technologies, the LC flow rates could be reduced to low nanoliter and to even picoliter scale to further enhance peptide separation resolution and ionization efficiency. MS instrumentation with high ion-transmission optics and sensitive detectors could provide further enhancements in proteome coverage for single cells. In addition to FAIMS, other ion mobility-based technologies, including trapped ion mobility spectrometry (TIMS) (Michelmann et al., 2015; Vasilopoulou et al., 2020) and, particularly, structures for lossless ion manipulation (SLIM), can offer improved ion separation and overall ion utilization efficiencies. With all these developments, we believe the proteome depths of scProteomics will reach the level of single-cell RNA sequencing and ultimately become an indispensable tool in biological and medical research.

## STAR★METHODS

### RESOURCE AVAILABILITY

#### Lead contact

Further information and requests for resources and reagents should be directed to and will be fulfilled by the lead contact, Ying Zhu (ying.zhu@pnnl.gov).

#### Materials availability

This study did not generate new unique reagents.

#### Data and code availability

The mass spectrometry proteomics data have been deposited to the ProteomeXchange Consortium via the MassIVE partner repository. The accession number and DOI are listed in the key resources table.

This paper does not report original code.

Any additional information required to reanalyze the data reported in this paper is available from the lead contact upon request.

### EXPERIMENTAL MODEL AND SUBJECT DETAILS

#### Cell culture

All cell lines used in this study were maintained in a medium compatible with each cell line and incubated at 37 °C with 5% of CO_2_. Of the three leukemia cell lines, K562 and MOLM14 cells were cultured in RPMI-1640 medium supplemented with 10% fetal bovine serum (FBS), and CMK cells were maintained in RPMI-1640 medium with 20% FBS added. For HeLa cells, DMEM supplemented with 10% FBS was added. RAW 264.7 cells were maintained in DMEM supplemented with 10% FBS followed to be stimulated with 100 ng/ul of LPS (Sigma Aldrich) in serum-free DMEM (Thermo Fisher Scientific) for 24 hr or 48 hr. For the control of RAW264.7 cells (non-treated), ten million cells were collected before stimulation with LPS. In the same way, LPS-stimulated cells were harvested after 24 hr or 48 hr of treatments.

#### Primary lung cells

The dissociated primary human lung cells was kindly provided by Dr. Gloria Pryhuber at University of Rochester Medical Center. The detailed protocol to generate the human lung cells was described previously (Bandyopadhyay et al., 2018) and available on protocol.io (https://doi.org/10.17504/protocols.io.biz5kf86). The dissociated lung cells in 90% FBS and 10% DMSO were cryo-frozen in −80°C freezer. A freezing vial was shipped to PNNL on dry ice.

### METHOD DETAILS

#### Single-cell sorting

HeLa and RAW 264.7 cells were washed by chilled PBS and sorted on the nanoPOTS chips (4 × 3 12, 1.2 mm diameter per well) using the Influx II cell sorter (BD Biosciences, San Jose, CA) as described previously (Zhu et al., 2018a). To build the in-depth spectral library, 50 cells of each cell line (or equivalent peptides of ∼10 ng) were loaded onto the microPOTS chip (3 × 9, 2.2-mm diameter per well). For primary lung cells, the cells were thawed and resuspended in DMEM with 10%FBS for 1 Hr prior to be centrifuged at 800 g for 10 min. The supernatant was removed and cells were washed in DPBS. To gate out dead cells or cell debris, the cells with labeled with Calcein AM viability dye (Thermo Fisher). Similar to the FACS-sorting procedures above, we sort 50 cells into microPOTS chips for library generation and single cells into nanoPOTS chips for analysis.

#### Protein digestion

For the low-input mock samples (0.2 ng, equivalent amount peptides to a single-cell), leukemia cell lines were lysed in a tube with lysis buffer including 50 mM NH_4_HCO_3_ (pH8.0), 8 M UREA, and 1 % phosphatase inhibitor followed by sonicated in a cold bath for 3 min. After the measurements of the protein concentrations by BCA assay (Thermo Fisher Scientific), proteins equivalent to 200 μg were reduced in 5 mM DTT for 1 hr at 37 °C and alkylated with 10 mM iodoacetamide (IAA) in the dark for 1 hr at room temperature. Eightfold diluted samples with 50 mM NH_4_ HCO_3_ were digested with Lys-C peptidase at 37 °C with a ratio of 50:1 (w/w) for 3 hr followed by digesting with trypsin with a ratio of 50:1 (w/w) at 37 °C overnight. The tryptic digested peptides were acidified by 0.5% trifluoroacetic acid (TFA) at final concentration, then desalted using C18 SPE tips. After concentrated, the BCA assay was performed to estimate the final concentration of the peptides. Using the nanoPOTS robot, 0.2 ng and 10 ng of the peptides from each leukemia cell line were loaded on the nanowell/microwell chips and completely dried by a vacuum system (Williams et al., 2020).

For single-cell analysis, single and 50 FACS-sorted cells on the chip were processed on the nanoPOTS platform for single cells and spectral library, respectively. To extract proteins, we first added a lysis buffer containing 0.2% n-Dodecyl b-D-maltoside (DDM) and 5 mM DTT in 0.5 × PBS and 25 mM NH_4_HCO_3_ buffer in each well, then incubated for 1 hr at 70 °C. Denatured and reduced proteins were alkylated with 10 mM IAA in the dark for 30 min at RT. Double enzymatic digestions were performed by incubating with LysC (1 ng for single-cell, 5 ng for 50 cells) for 4 hr at 37 °C followed by treatment with trypsin (2 ng for single-cell, 10 ng for 50 cells) overnight. Peptides were acidified with 5% formic acid and completely dried using a vacuum system. All chips were stored in a −20 °C freezer until MS analysis.

*Shewanella oneidensis* MR-1 peptide was obtained from a non-related study. The sample preparation procedures were described in detail previously (Xiang et al., 2020; Zhu et al., 2018c).

#### LC-FAIMS-MS/MS analysis

The in-house assembled nanoPOTS autosampler contains an in-house packed SPE column (100 μm i.d., 4 cm, 5 μm, 300 Å C18 material, Phenomenex) and an LC column (50 μm i.d., 25 cm long, in-house packed with 1.7 μm, 190 Å C18 material, Waters) using a self-pack picofrit bare column (cat. no. PF360–50-10-N-5, New Objective, Littleton, MA). The LC column is heated to 50 °C using Agile-Sleeve column heater (Analytical Sales and services, Inc., Flanders, NJ) for sample analysis (Williams et al., 2020). Briefly, samples were dissolved with Buffer A (0.1% formic acid in water) on the chip, then trapped on the SPE column for 5 min. After washing the peptides, samples were eluted at 100 nL/min and separated using a 60-min gradient from 8% to 35% Buffer B (0.1% formic acid in acetonitrile).

An Orbitrap Fusion Lumos Tribrid MS (Thermo Scientific) with FAIMSpro operated in data-dependent acquisition mode was used for all analyses. Peptides were ionized by applying a voltage of 2,000 V or 2,400 V for standard or FAIMS methods, respectively.

For the standard method, precursor ions with mass range 375–1600 m/z were scanned at 120,000 resolution with an ion injection time (IT) of 254 ms and an AGC target of 1E6. To analyze pooled samples for generating the spectral libraries, the selected precursor ions with +2 to +7 charges were fragmented by a 30% level of high energy dissociation (HCD) and scanned at 60,000 resolution with an IT of 118 ms and an AGC target of 1E5. When single-cell level (0.2 ng) peptides were injected, fragmented peptide ions were scanned at 120,000 resolution with an IT of 246 ms and an AGC target of 1E5.

For the TIFF method, the ionized peptides were fractionated by the FAIMSpro interface using a 2-CV (−45, −65 V) method or a 4-CV (−45, −55, −65, −75 V) method. Fractionated ions with a mass range 350–1500 m/z were scanned at 120,000 resolution with an IT of 254 ms and an AGC target of 1E6. For the pooled samples for generating a spectral library, a single CV was used for each LC-MS run. Precursor ions with intensities > 1E4 were selected for fragmentation by 30% HCD and scanned in an ion trap with an AGC of 2E4 and an IT of 150 ms. For single-cell samples, cycle times of 1.5 s and 0.6 s were used for the 2-CV and 4-CV methods, respectively. Precursor ions with intensities > 1E4 were fragmented by 30% HCD and scanned with an AGC of 2E4 and an IT of 254 ms.

### QUANTIFICATION AND STATISTICAL ANALYSIS

All raw files were processed by MaxQuant (Ver. 1.6.2.10) with the Uniport protein sequence database of *homo sapiens* (Proteome ID: UP000005640; Downloaded in 03/12/2020 containing 20,364 reviewed sequences) and of *mus musculus* (Proteome ID: UP000000589; Downloaded in 5/19/2020 containing 17,037 reviewed sequences) using the Andromeda search engine with a 6-ppm precursor ion tolerance after mass calibration (Tyanova et al., 2016a). Protein acetylation in N-terminal and oxidation at methionine were chosen as variable modifications. Carbamidomethylation of cysteine residues was set as a fixed modification. Both proteins and peptides were filtered with a false discovery rate (FDR) less than 0.01. Match between runs algorithm in Maxquant was activated with a matching window of 0.4 min and alignment windows of 10 min. For raw files with multiplex FAIMS CVs, we converted them to multiple mzxml files corresponding to separate individual CVs using an in-house converting tool (https://github.com/PNNLComp-Mass-Spec/FAIMS-MzXML-Generator/releases). Those separated files were assigned to non-adjacent fractionation numbers (e.g., 1, 3, 5, 7) during the Maxquant search to ensure feature matching only occurs between the files with the same CV.

It should be noted that Fragpipe (V16.0 or higher) (https://github.com/Nesvilab/FragPipe) has supported the direct analysis of FAIMS datasets and integrated the three-dimensional feature marching algorithm (Kong et al., 2017; Yu et al., 2021).

For label-free quantification of single-cell-level peptides (0.2 ng) for three leukemia cell lines and dissociated human lung single-cell, Perseus (Ver. 1.6.12.0) was utilized for the data clean and statistical analysis (Tyanova et al., 2016b). The iBAQ algorithm was used for the single-cell analysis because the iBAQ values are proportional to the molar quantities of the proteins. We log_2_ transformed the iBAQ values after filtering out contaminants and reverse identifications. Missing values were imputed based on a standard distribution (width: 0.3, downshift: 1.8) to simulate signals for low-abundance proteins. Data were normalized using width adjustment, which subtracts medians and scales for all values in a sample to show equal interquartile ranges. Two-way t-tests were performed for the pairwise comparison of the leukemia cell lines proteomes utilizing the threshold of Benjamini-Hochberg FDR < 0.05 and S_0_=0.1, while ANOVA tests were employed for multiple sample tests of dissociated human lung single cells with Permutation based FDR < 0.05. To clarify cell populations from dissociated lung cells, multiple steps including principal components analysis (PCA) and hierarchical clustering were employed using Perseus. Gene ontology analysis for the biological process of the molecules was performed in DAVID web-based bioinformatic tools (database version 6.8, https://david.ncifcrf.gov/) (Huang et al., 2009).

The processing of the macrophage single-cell data was performed using an R package; RomicsProcessor v1.1.0 (https://github.com/PNNL-Comp-Mass-Spec/RomicsProcessor). Briefly, the “proteingroups.txt” output file of the MaxQuant search was imported as a multilayered R object with its associated metadata to extract iBAQ values of the identified proteins. The iBAQ values were then log_2_ transformed and filtered to allow maximal missingness of 50% within at least one given condition. After median normalization, batch correction was applied to remove the batch effects between chips using ComBat algorithm from the SVA package (v3.36.0). The missing values were imputed using the function of imputeMissing() and UMAP (the uniform manifold approximation and projection)-based dimensional reduction analysis was performed using the romicsUmapPlot() function in the RomicsProcessor package. For the statistics, ANOVA test was applied with a Benjamini-Hochberg FDR < 0.001 and a S_0_=5; we applied a highly significant level to a large number of macrophage cells data in which the group was clearly distinguished by the duration of LPS treatment to give a statistical role to the difference between the median value.

## Supplementary Material

1

4

3

2

5

## Figures and Tables

**KEY RESOURCES TABLE:** 

REAGENT or RESOURCE	SOURCE	IDENTIFIER

Biological samples		

Primary human lung cells	University of Rochester Medical Center	Donor D011, Provided by Dr.Gloria Pryjuber

Chemicals, peptides, and recombinant proteins		

Fetal bovine serum	Thermo Fisher Scientific	10–082-147
RPMI-1641	Thermo Fisher Scientific	11875093
Dulbecco’s Modified Eagle Medium (DMEM)	Thermo Fisher Scientific	11965092
Lipopolysaccharides (LPS) from *Escherichia coli*	Sigma Aldrich	L2630–10MG
Calcein AM	Thermo Fisher Scientific	C3100MP
UREA	Sigma Aldrich	U5128
Ammonium bicarbonate (NH_4_HCO_3_)	Sigma Aldrich	S2454
Dithiothreitol (DTT) No-Weigh™	Thermo Fisher Scientific	A39255
Iodoacetate (IAA), Single-Use	Thermo Fisher Scientific	A39271
Formic acid, LC-MS grade	Thermo Fisher Scientific	28905
Lys-C, Mass Spectrometry Grade	Promega	V1671
Trypsin, Mass Spectrometry Grade	Promega	V5280
n-Dodecyl β-D-maltoside (DDM)	Sigma Aldrich	D4641–1G
10x phosphate buffered saline (PBS)	Sigma Aldrich	P5493–1 L

Critical commercial assays		

Pierce™ BCA Protein Assay Kit	Thermo Fisher Scientific	23225

Deposited data		

Proteomics RAW files	MassIVE	MSV000085937; https://doi.org/10.25345/C5PR1P

Experimental models: Cell lines		

K-562 human cell line	Oregon Health & Science University	Provided by Dr.Anupriya Agarwal, originally obtained from ATCC (CCL-243)
MOLM-14 human cell line	Oregon Health & Science University	Provided by Dr.Anupriya Agarwal, originally established from the peripheral blood of a patient at relapse of acute monocytic leukemia by Dr. Matsuo et al. at Fujisaki Cell Center in Japan
CMK human cell line	Oregon Health & Science University	Provided by Dr.Anupriya Agarwal, originally obtained from the German National Resource Center for Biological Material
RAW 264.7 mouse cell line	ATCC	TIB-71
HeLa human cell line	ATCC	CCL-2

Software and algorithms		

MaxQuant (Ver 1.6.2.10)	Max Planck Institute of Biochemistry	https://www.maxquant.org/
Perseus (Ver 1.6.12.0)	Max Planck Institute of Biochemistry	https://www.maxquant.org/perseus/
FAIMS MzXML converting tool	PNNL	https://github.com/PNNL-Comp-Mass-Spec/FAIMS-MzXML-Generator/releases
RomicsProcessor R package	PNNL	https://github.com/PNNL-Comp-Mass-Spec/RomicsProcessor
GraphPad Prism Ver.8.3.0	GraphPad Software	https://www.graphpad.com/scientific-software/prism/

## Highlights

- TIFF is an ion-mobility-based method for data acquisition and peptide identification
- Three-dimensional feature matching increases sensitivity and reduces false discovery
- TIFF method enables unbiased proteome analysis to a depth of ~1,100 proteins per cell
- Single-cell proteome changes during macrophage activation were studied
