# Supplementary material for: Three-dimensional feature matching improves coverage for single-cell proteomics based on ion mobility filtering
Source: Cell Syst. Author manuscript; Available in PMC 2022 May 20. (PMC9119937; doi:10.1016/j.cels.2022.02.003)
Supplement: 1 [file NIHMS1791064-supplement-1.pdf]

Cell Systems, Volume *13*

Supplemental information

Three-dimensional feature matching improves coverage for single-cell proteomics based on ion mobility filtering

Jongmin Woo, Geremy C. Clair, Sarah M. Williams, Song Feng, Chia-Feng Tsai, Ronald J. Moore, William B. Chrisler, Richard D. Smith, Ryan T. Kelly, Ljiljana Paša-Tolić, Charles Ansong, and Ying Zhu

Cell Systems, Volume *13*

Supplemental information

Three-dimensional feature matching improves coverage for single-cell proteomics based on ion mobility filtering

Jongmin Woo, Geremy C. Clair, Sarah M. Williams, Song Feng, Chia-Feng Tsai, Ronald J. Moore, William B. Chrisler, Richard D. Smith, Ryan T. Kelly, Ljiljana Pasa-Tolic, Charles Ansong, and Ying Zhu

Supplementary Information for

Three-dimensional feature matching improves coverage for single-cell proteomics based on ion mobility filtering

Jongmin Woo,[1,4] Geremy C. Clair,[2,4] Sarah M. Williams,[1] Song Feng,[2] Chia-Feng Tsai,[2] Ronald J. Moore,[2] William B. Chrisler,[2] Richard D. Smith,[2] Ryan T. Kelly,[1,3] Ljiljana Pasa-Tolic,[1] Charles Ansong,[2] Ying Zhu[1,5,]*

[1]Environmental Molecular Sciences Laboratory, Pacific Northwest National Laboratory, Richland, Washington 99354, United States

[2]Biological Sciences Division, Pacific Northwest National Laboratory, Richland, Washington 99354, United States

[3]Department of Chemistry and Biochemistry, Brigham Young University, Provo, Utah 84604, United States

[4]These authors contributed equally

[5]Lead contact

*Correspondence: ying.zhu@pnnl.gov

Supplementary Materials

(a)

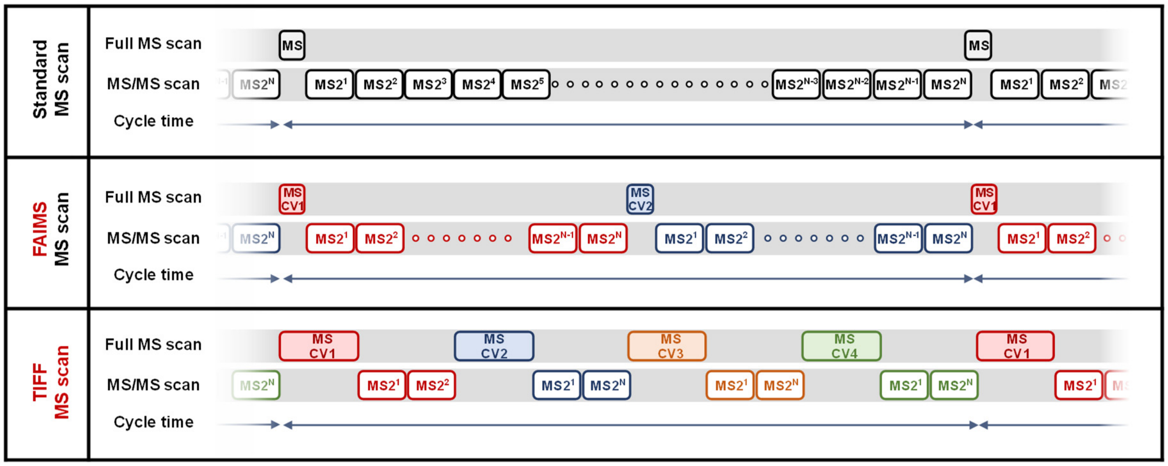

(b)

	FAIMS	TIFF
LC gradient (min)	160	80
Flow rate (nL/min)	20	100
# of CVs	2	4
Cycle time per CV (s)	1.5	0.8
# of MS1	7198	7200
# of MS2	21345	3098
Percentage of MS1 acquisition time (%)	20.58	69.90

(c)

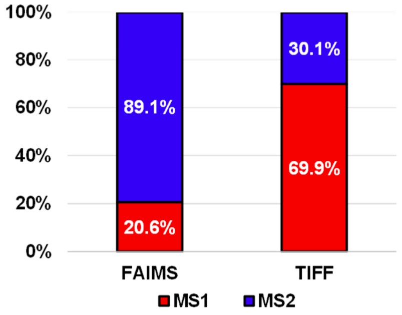

Figure S1. Comparison of different MS acquisition methods, related to Figure 1. (a) Schematic illustration of three different MS acquisition methods. (Upper) standard MS method without FAIMS; (Middle) standard FAIMS-MS method; (Bottom) The transferring identification based on FAIMS filtering (TIFF) method. In the TIFF method, the elongated ion accumulations for MS1 scan can increase the sensitivity of MS1-level peptide detection. The peptide features are identified by matching to a spectral library based on 3D tags (LC retention time, accurate m/z, and FAIMS CV). Small number of MS/MS scans are used for non-linear alignment during MaxQuant search. **(b)** The comparison of standard FAIMS(Cong et al., 2021) with the TIFF method for single-cell proteomics. **(c)** The normalized MS acquisition time for MS1 and MS2 scans. The precursor ion sampling efficiency of the TIFF method is increased by > 2 folds compared with the previous FAIMS method (Cong *et al.*, 2021).

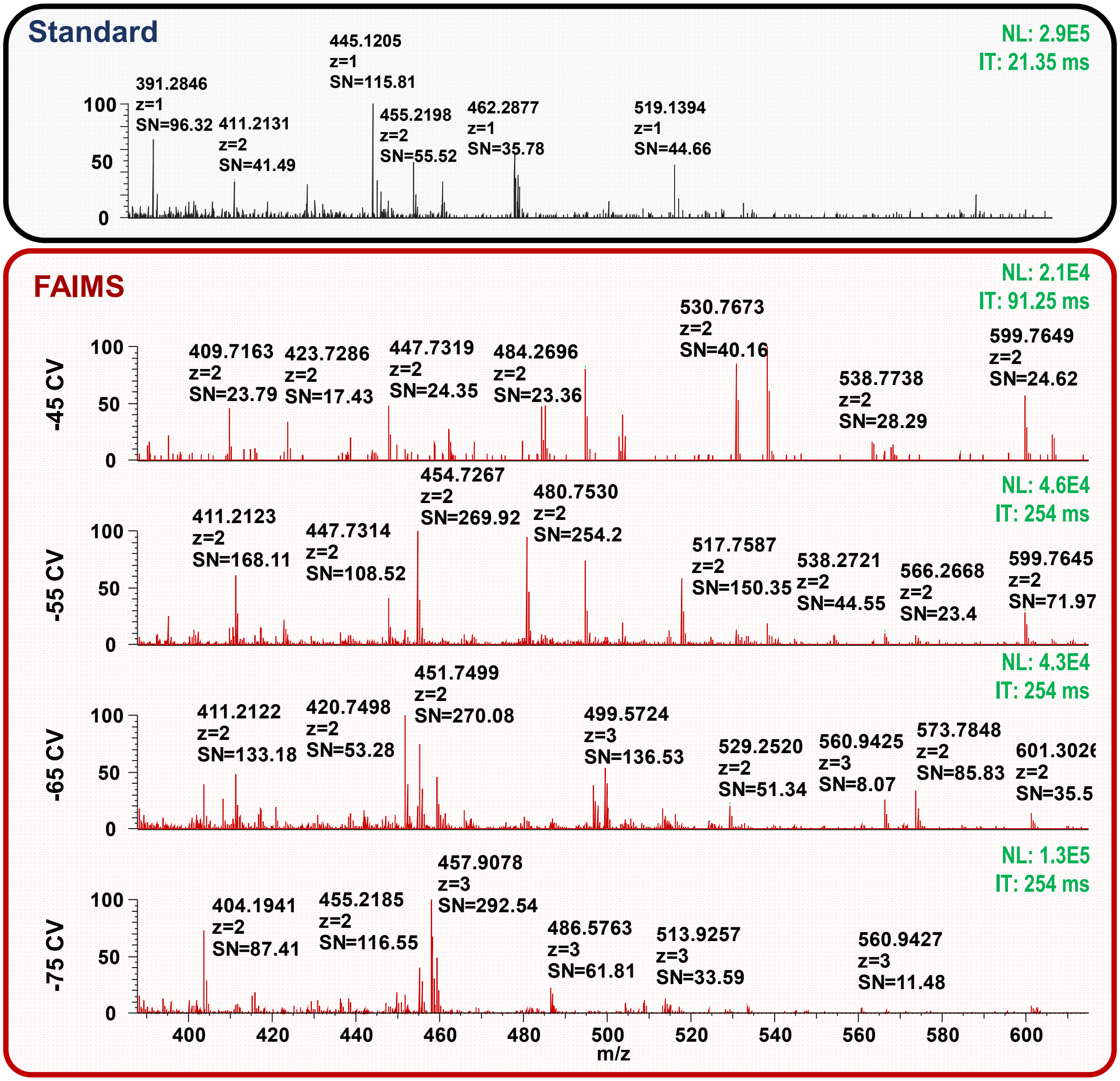

Figure S2. Representative spectra are chosen from the MS raw files of a standard method (in the blue box) and a FAIMS method with 4 CVs (in the red box), related to Figure 1. The spectra are extracted from a similar retention time. Spectra are labeled with m/z, ion charge state, and signal to noise (SN) ratio.

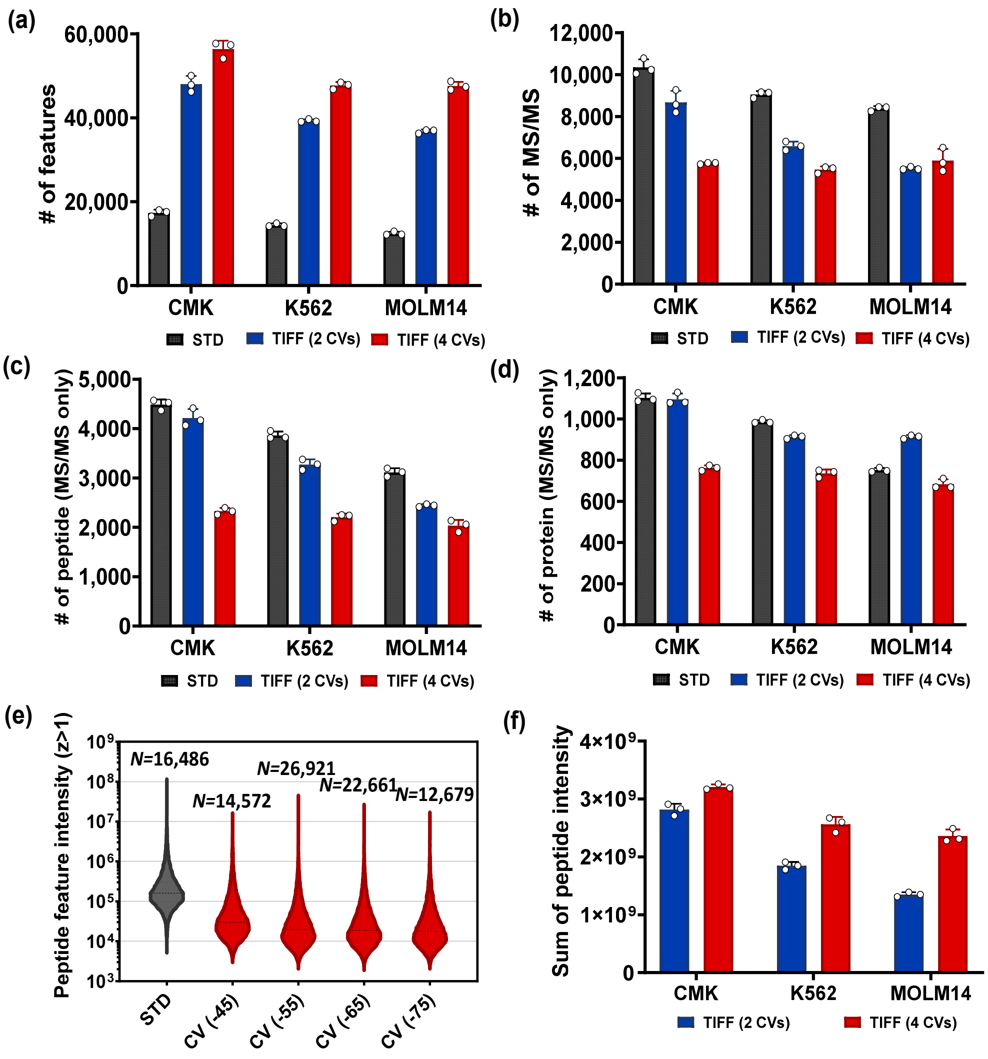

Figure S3. The comparison of detection sensitivity for different MS acquisition methods, related to Figure 1. **(a-d)** Benchmarking of the standard, 2-CV-TIFF, and 4-CV TIFF methods using single-cell level peptides (0.2 ng) from three cell lines (CMK, K562, and MOLN14). **(a)** The number of peptide features (charge>+1); **(b)** MS/MS scans; and **(c)** unique peptides; **(d)** proteins identified by MS/MS. **(e)** Intensity distributions of peptide features (z > +1) obtained by the standard and 4-CV-TIFF methods using 0.2-ng CMK peptides. Labeled numbers indicate the numbers of detected peptide features. An in-house MASIC tool was used to select the peptide features from MSGF+ results. The numbers (*n*) of datapoints are annotated above each violin plot. **(f)** The summed peptide intensities from the 2-CV and 4-CV TIFF methods. For a, b, c, d, and f, the data point (*n*) to generate the bar graphs is 3. Error bars indicate standard deviations.

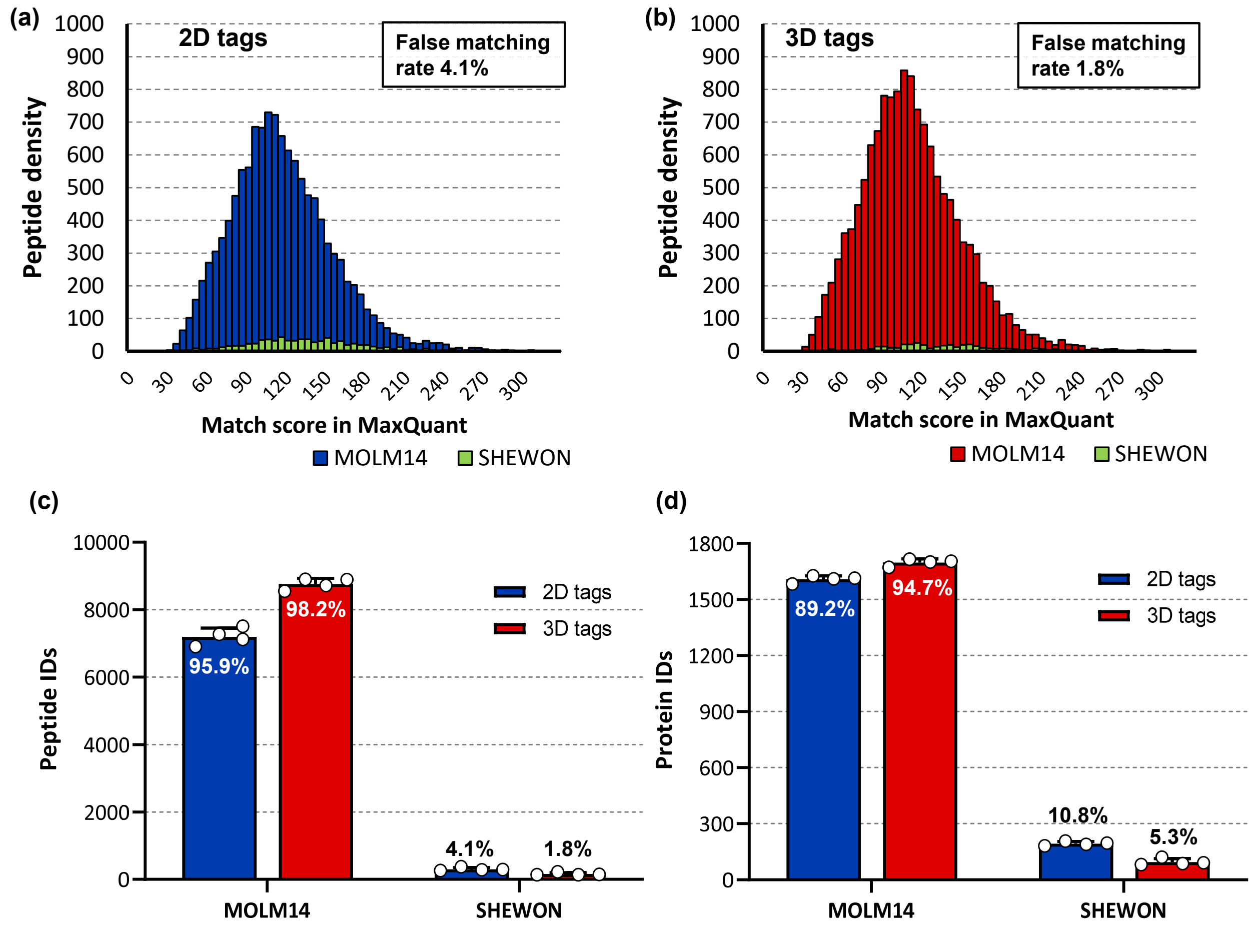

Figure S4. Evaluation of the false matching rates by matching a human sample to a mixed-species spectral library containing 20588 human peptides from MOLM14 cells and 9362 bacterial peptides from Shewanella Oneidensis MR-1, related to Figure 1. Histogram of the number of identified peptides with **(a)** two-dimensional tags (m/z and RT), or **(b)** three-dimensional tags, (m/z, RT, and FAIMS CV). **(c)** False discovery rates at the peptide and **(d)** protein levels using 2D or 3D matching approaches. The data point (*n*) to generate the bar graphs is 4.

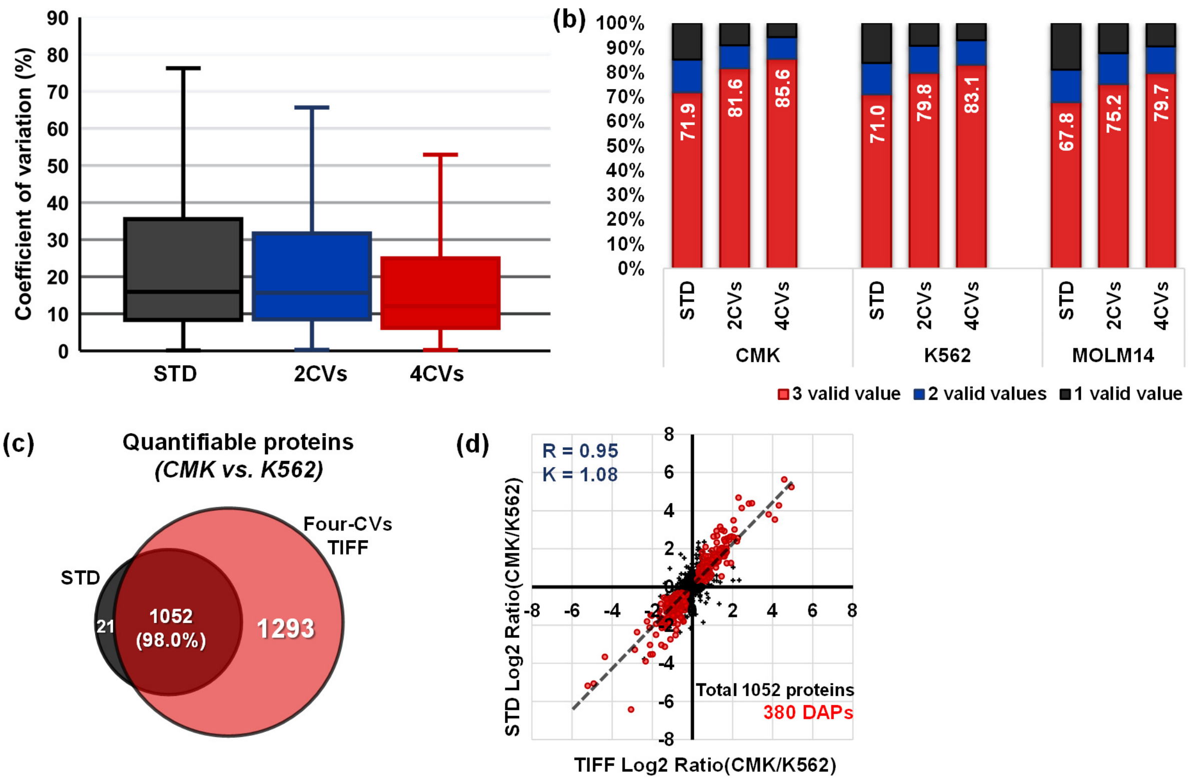

Figure S5. The evaluation of quantification performance of the TIFF method, related to Figure 1. (a) Distributions of the coefficient of variations for quantified proteins using the three MS acquisition methods (STD, 2-CV-TIFF, and 4-CV-TIFF. Coefficient of variation plot: *n*=1,146 for STD, *n*=2,353 for 2CVs, and *n*=2,634 for 4 CVs datasets). **(b)** Percentage of valid values using the three methods. Red, blue, and black colors indicate the percentages of proteins with valid values of 3, 2, and 1 across the triplicate, respectively. **(c)** Overlap of quantifiable proteins between CMK and K562 samples measured by standard and 4-CV-TIFF methods. **(d)** The linear correlation of log2-transformed fold changes of CMK and K562 cells between the TIFF method (4 CVs) and the STD method. Red dots indicate differentially abundant proteins (DAPs) in both methods calculated by t-test (FDR<0.05, S_0=0.1).

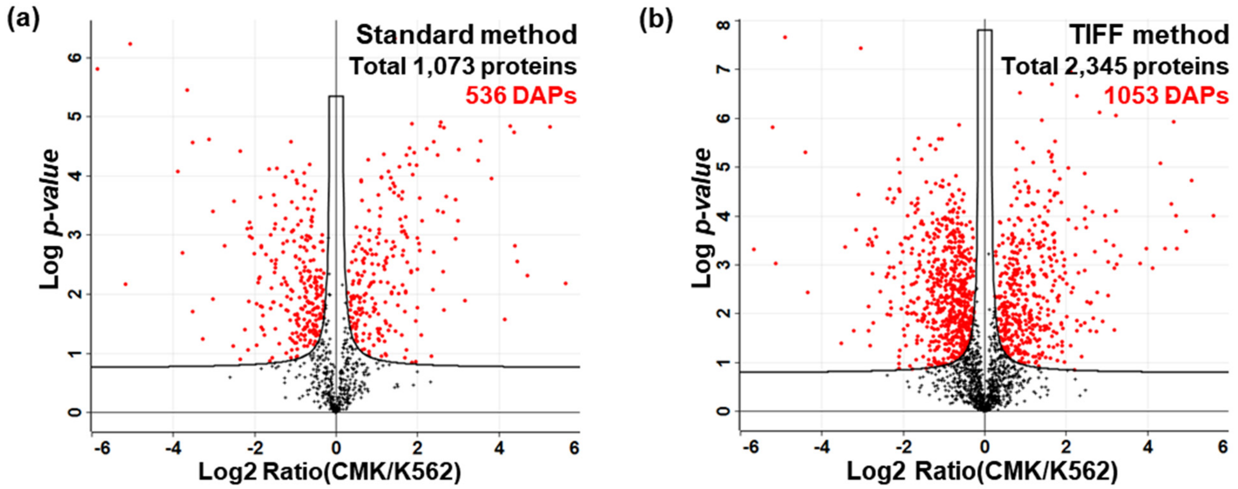

Figure S6. Differentially abundant proteins between CMK and K562 cell lines, related to Figure 1. (a-b) Statistics analysis to identify differentially abundant proteins (DAPs) between CMK and K562 cells using iBAQ intensities (t-test FDR < 0.05 and $S_0 = 0.1$). Volcano plots for **(a)** standard method and **(b)** the 4-CV TIFF method. Total quantified proteins and DAPs were labeled with red color.

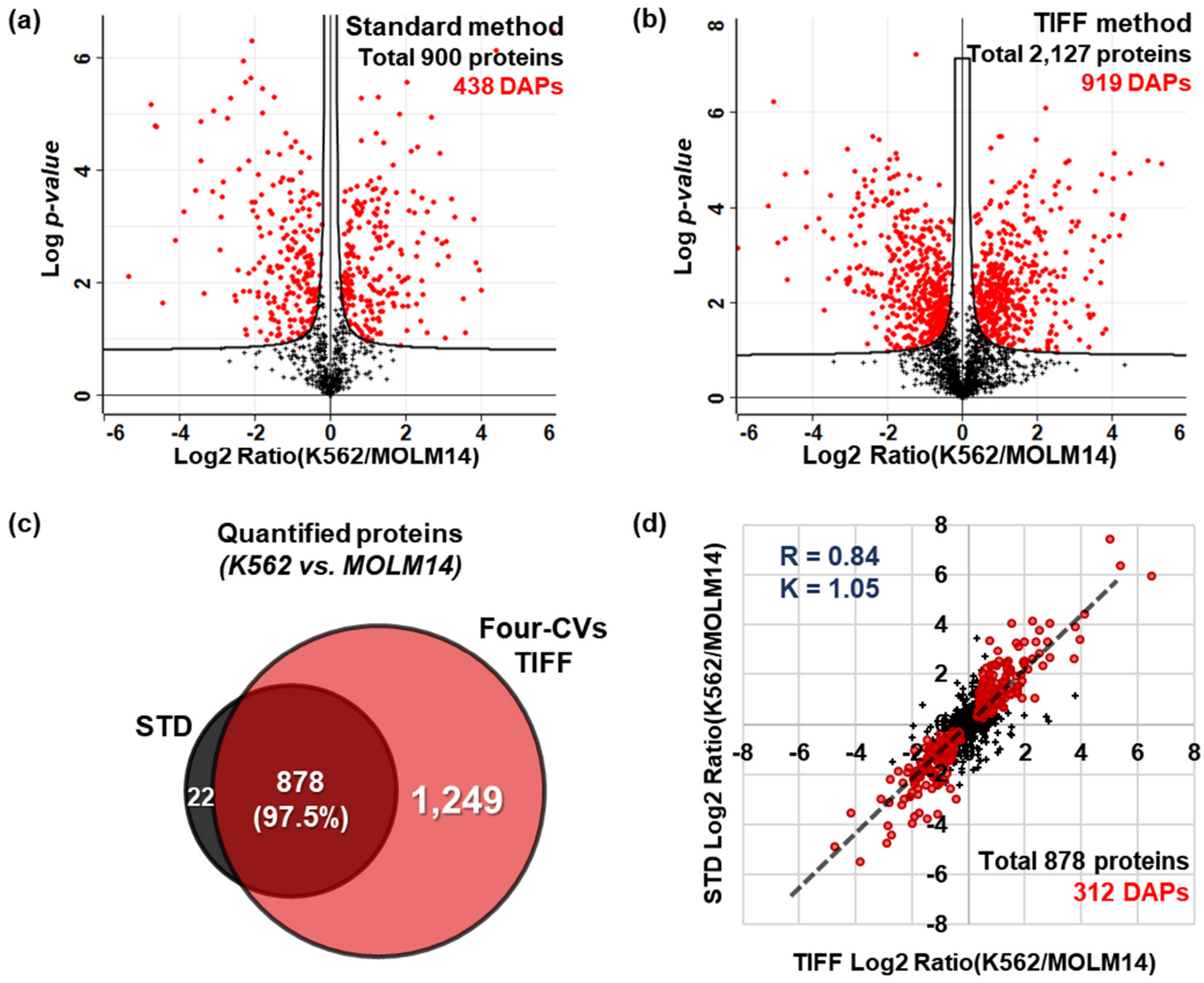

Figure S7. Differentially abundant proteins between K562 and MOLM14 cell lines, related to Figure 1. **(a-b)** Statistics analysis to identify differentially abundant proteins (DAPs) between K562 and MOLM14 cells (t-test FDR < 0.05 and $S_0 = 0.1$). Volcano plots for **(a)** the standard and **(b)** 4-CV TIFF methods. **(c)** Overlap of quantifiable proteins between K562 and MOLM14 cells measured by standard and TIFF methods (4 CVs). **(d)** The linear correlation and slope of log2 transformed fold changes of K562 and MOLM14 proteins between the 4-CV TIFF and STD methods. Red dots indicate DAPs in both methods calculated by t-test (FDR<0.05, S_0=0.1).

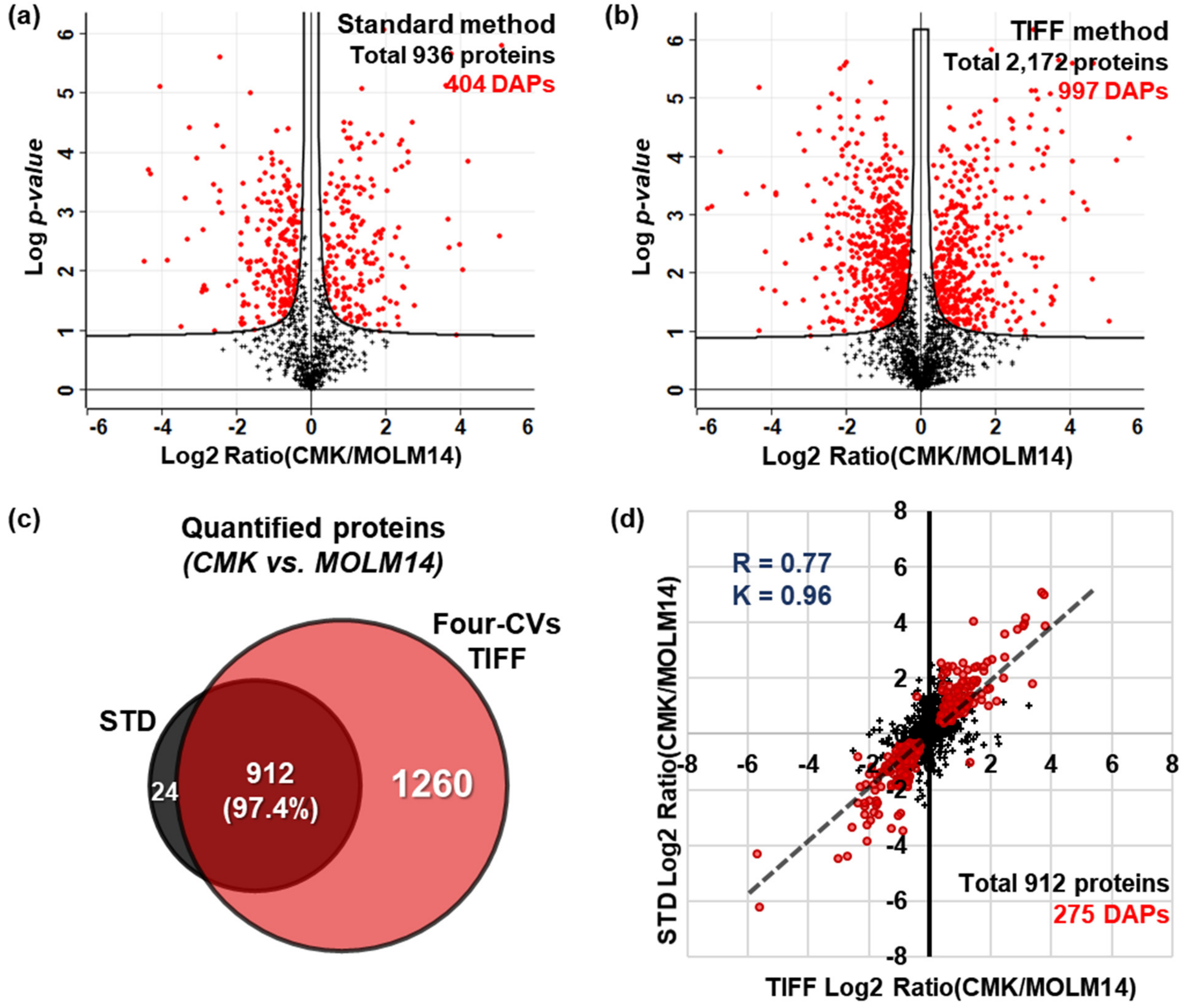

Figure S8. Differentially abundant proteins between CMK and MOLM14 cell lines, related to Figure 1. (a-b) Statistics analysis to identify differentially abundant proteins (DAPs) between CMK and MOLM14 cells (t-test FDR < 0.05 and $S_0 = 0.1$). Volcano plots for **(a)** the standard and **(b)** 4-CV TIFF methods. **(c)** Overlap of quantifiable proteins between CMK and MOLM14 cells measured by the standard and 4-CV TIFF methods. **(d)** The linear correlation of log2-transformed fold changes of CMK and MOLM14 proteins between the 4-CV TIFF and STD methods. Red dots indicate (DAPs) calculated by t-test (FDR<0.05, S_0=0.1).

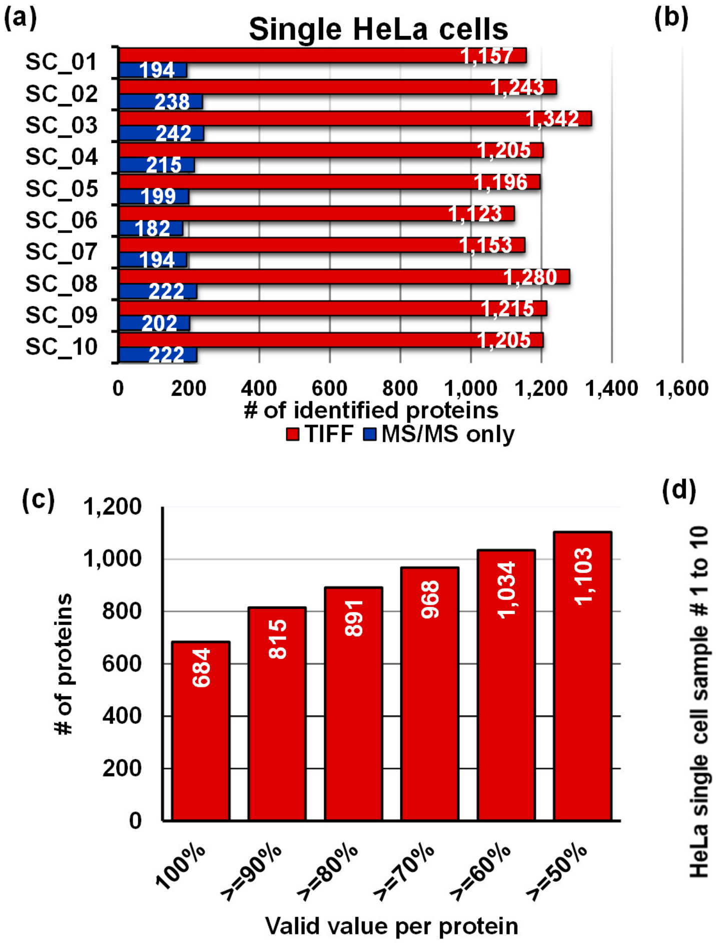

Figure S9. ScProteomics of HeLa cells using TIFF method, related to Figure 1. (a) Number of protein groups in single HeLa cells identified by MS/MS only (blue) and by the 4-CV TIFF method (red). **(b)** The overlap of identified proteins in single HeLa cells obtained in this study and a previous study with a similar LC-MS setting but without FAIMS (Zhu et al., 2018). **(c)** The numbers of proteins having valid values from 50% to 100% across the 10 single cells. **(d)** Pair-wise correlations of protein iBAQ intensities between the 10 cells. Proteins containing >70% valid values were required.

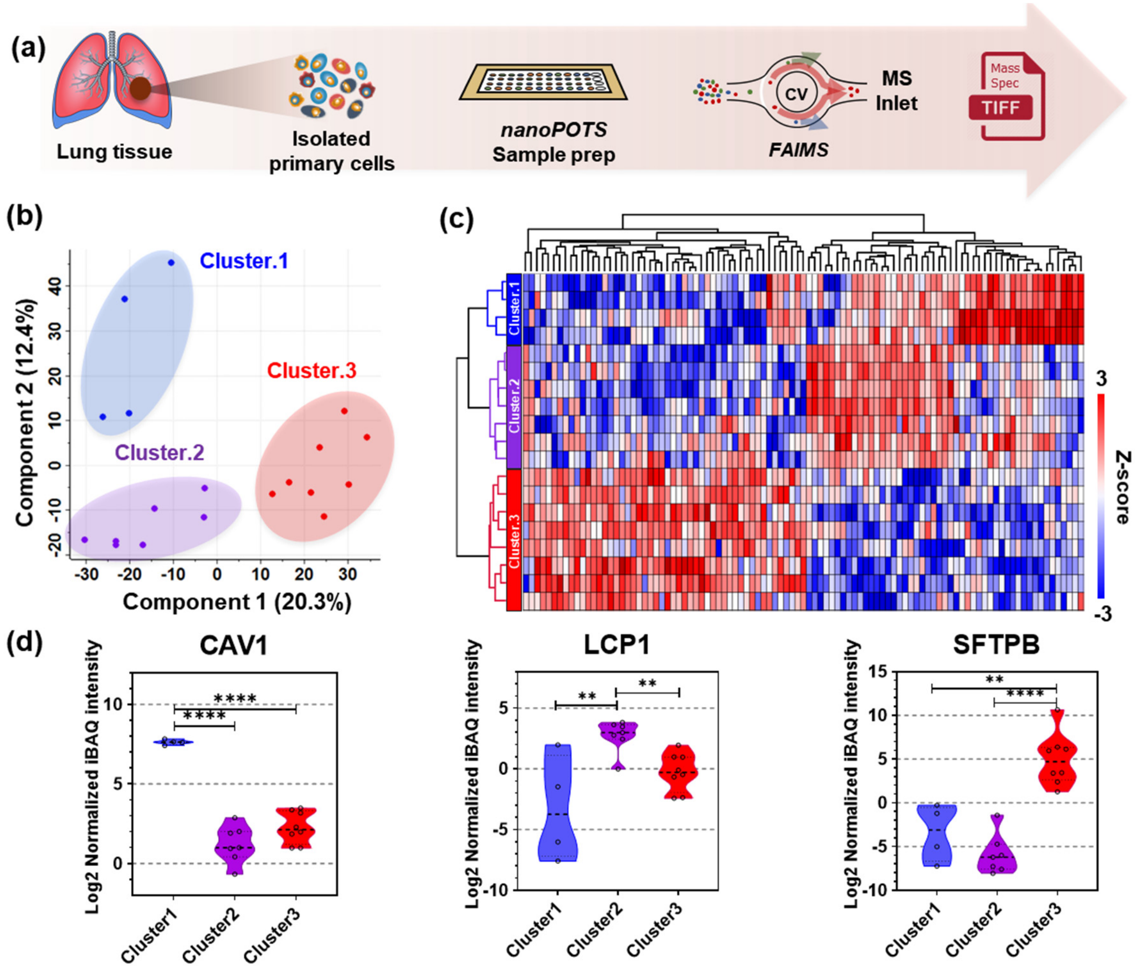

Figure S10. ScProteomics for classifying cell populations of a human lung, related to Figure 1. (a) Schematic workflow for scProteomic analysis of dissociated human lung tissue from a 2-year old donor. The tissue was dissociated into the single cells followed by applying the scProteomics pipeline including FACS isolation, nanoPOTS processing, autosampler-LC, and TIFF MS method. **(b)** PCA plot of un-defined cell types. **(c)** Heatmap of differentially abundant proteins (DAPs) by ANOVA test. **(d)** Representative proteins of putative cell types. Cluster 1, 2, and 3 were predicted as lung endothelial, immune, and epithelial cells, respectively (**<0.01, ****<0.0001). The numbers (*n*) of *iBAQ* intensity datapoints are 4 in cluster 1, 7 in cluster 2, and 8 in cluster 3 datasets.

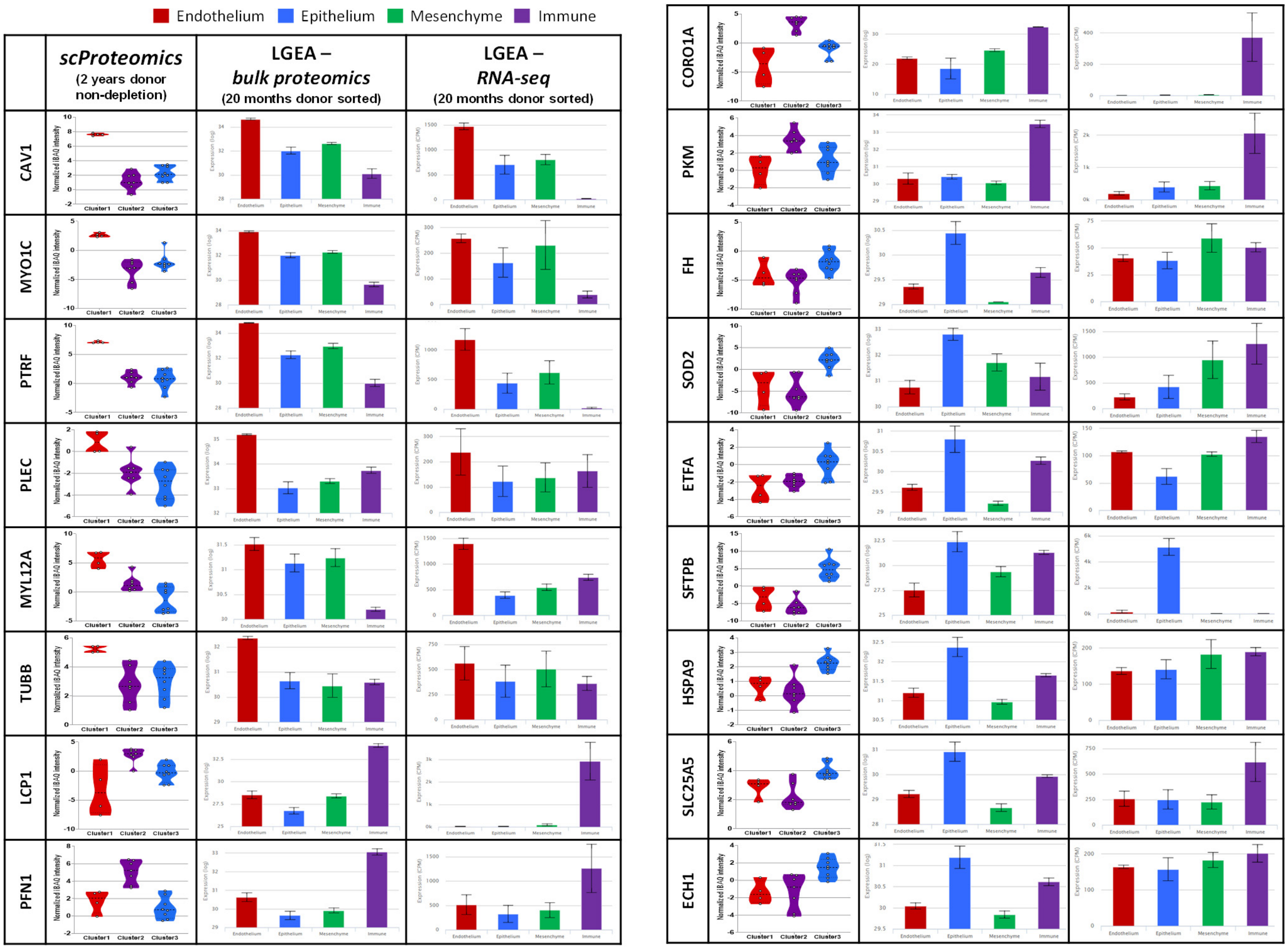

Figure S11. The abundance distributions of representative proteins markers in the scProteomics data and lung gene expression analysis (LGEA) database (https://research.cchmc.org/pbge/lunggens/mainportal.html) containing sorted human lung endothelial, epithelial, immune and mesenchymal cells measured by bulk proteomics and RNA sequencing (Du et al., 2019), related to Figure 1.

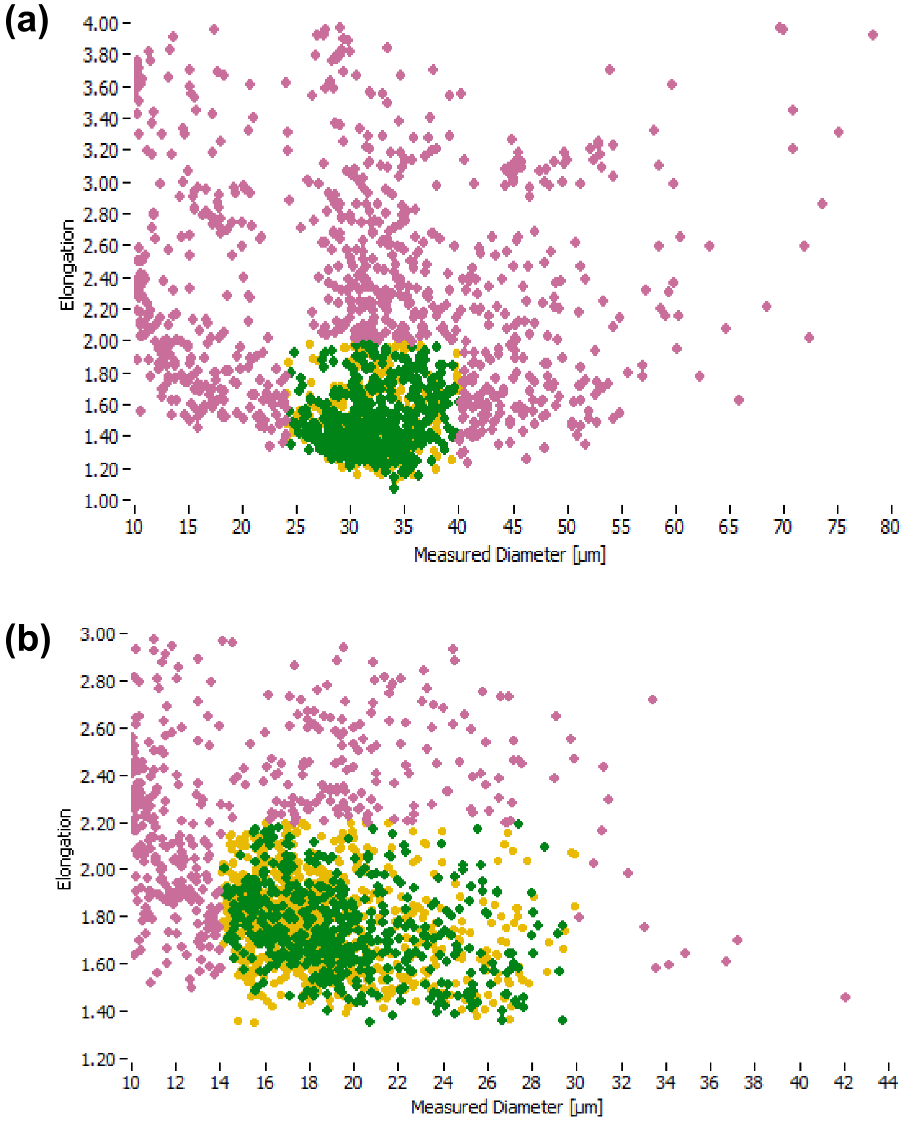

Figure S12. The size distributions for SVEC cells (a) and primary lung cells (b). The data was generated by CellenONE cell sorting system. Note the measured diameters are larger than the actual sizes due to the lens effect of piezo dispense capillary.

Table S1. Numbers of identified proteins in single mammalian cells from previously published papers using nanoPOTS and label-free analysis, related to Figure 1. Proteins were identified by MS/MS or by matching between runs (MBR) algorithm in MaxQuant software (Tyanova et al., Nat. Protoc. 2016) (MQ: MaxQuant, PD: Proteome Discoverer).

Single-cell approach	LC system (column I.D. in µm, flow rate in nL/min)	MS instrument	Cell Type	Protein Groups (by MS/MS)	Proteins Groups (MBR)	Reference
nanoPOTS	30 / 50	Lumos	HeLa	211	669	Zhu *et al.* Angew. Chem. (2018)
nanoPOTS with autosampler	50 / 150	Lumos	MCF10	250	773	Williams et al. Anal. Chem. (2020)
nanoPOTS with narrow-bore LC	20 / 20	Eclipse	HeLa	362	874	Cong et al. Anal. Chem. (2020)
nanoPOTS with FAIMS	20 / 20	Eclipse	HeLa	683 (By MQ)/ 1056 (By PD)	1475	Cong *et al.* Chem. Sci. (2021)
nanoPOTS with TIFF	50 / 100	Lumos	HeLa	209	1212	*This study*

Table S2. A list of identified proteins from 19 single lung cells, related to Figure 1.

Table S3. A list of 402 quantifiable proteins of 19 single lung cells, related to Figure 1.

Table S4. A list of statistically significantly abundant proteins classifying three cell populations, related to Figure 1.

Table S5. The source datasets of 155 RAW 264.7 single cells with iBAQ intensities, related to Figure 2.
